# Effect of transparent substrate on properties of CuInSe_2_ thin films prepared by chemical spray pyrolysis

**DOI:** 10.1038/s41598-022-18579-w

**Published:** 2022-08-30

**Authors:** Maryam Hashemi, Zahra Saki, Mehdi Dehghani, Fariba Tajabadi, Seyed Mohammad Bagher Ghorashi, Nima Taghavinia

**Affiliations:** 1 Department of Laser and Photonics, University of Kashan, Kashan, PO Box 873175-3153, Iran; 2 Department of Physics, Sharif University of Technology, Tehran, 14588 Iran; 3 Department of Nanotechnology and Advanced Materials, Materials and Energy Research Center, Karaj, PO Box 31787-316, Iran

**Keywords:** Materials science, Nanoscience and technology, Optics and photonics, Physics

## Abstract

In this paper, the properties of CuInSe_2_ (CISe) films deposited on three transparent substrates (FTO, FTO/NiO_x_, FTO/MoO_3_) are studied. These substrates might be used for bifacial solar cells, in place of the conventional glass/Mo substrates. CISe layers are deposited by spray pyrolysis followed by a selenization process. For the same deposition conditions, the CISe layers on FTO show the largest grain size (~ 0.50 µm) and crystallinity, while FTO/MoO_3_ substrates result in the smallest grains (~ 0.15 µm). The optical bandgap of the CISe films ranged from 1.35 eV for FTO substrate to 1.44 eV for FTO/MoO_3_ substrate. All films show p-type conductivity, with the carrier densities of 1.6 × 10^17^ cm^−3^, 5.4 × 10^17^ cm^−3^, and 2.4 × 10^19^ cm^−3^ for FTO, FTO/NiO_x_, and FTO/MoO_3_ substrates, respectively. The CISe films also show different conduction, and valence levels, based on the substrate. In all cases, an ohmic behavior is observed between the CISe and substrate. The results demonstrate that CISe layer crystallinity, carrier concentration, mobility, and energy levels are strongly dependent on the chemical nature of the substrate. Bare FTO shows the most appropriate performance in terms of device requirements.

## Introduction

Ternary semiconductors such as thin films of CuInGaSe_2_ (CIGS), CuInSe_2_ (CISe), CuInS_2_ (CIS), and their alloys have been widely used as absorbers for thin-film solar cells with high efficiencies and long-time stability. They have a narrow bandgap, high absorption coefficients, and good carrier-transport properties^1,2^. Recently these materials have shown successful application in other fields including a hole transporting material in perovskite solar cells^3,4^, photoelectrochemical water splitting^5,6^, photodetectors^7,8^, and photo-catalyst^9,10^. Although it has been a long time since the introduction of this class of semiconductors, it is still a challenge to make a low-cost film with proper features especially with the application in photovoltaics (PVs).

The deposition of CIGS and CISe thin films can be made using vacuum or solution-based techniques. The usual vacuum methods such as evaporation and/or sputtering are expensive and require high energy input. Therefore, alternative approaches such as solution-based methods have been studied. Solution-based methods include spin coating^11^, spray pyrolysis^12,13^, solvothermal^14^, electrodeposition^15–17^, printing^18^, successive ionic layer adsorption and reaction (SILAR)^19^ and colloidal methods^6,20^. Among all inexpensive techniques, spray pyrolysis is one of the best methods to deposit low-cost, highly scalable, and suitable CISe thin films for roll-to-roll production^12,13,21^.

However, due to the complex nature of CISe, a wide range of parameters needs to be controlled to get a suitable film in PV applications. Studying CISe films deposited at various conditions on different substrates shows that considerable parameters such as growth methods, thermal annealing, substrate temperature, thickness, composition, and adhesion can affect the grain structure, defect states, orientation texture of the CISe films, and hence the PV device performance^22–25^.

Despite a considerably complicated and expensive deposition process, metallic molybdenum (Mo), is considered as the conventional opaque substrate due to thermal and mechanical stability, low resistance, excellent adhesion with the substrate and the absorber, low film stress, and high optical reflectance^26–29^. Utilizing a transparent substrate is beneficial in the sense that leads to bifacial photovoltaic devices. Transparent substrates can be used to make PV devices since in this case light can penetrate through the entire device structure both from the front and the back contacts at different times of the day, leading to the creation of more photo-generated charge carriers^30–32^.

The properties of deposited CIGS and/or CISe films by various deposition methods on transparent electrodes have already been reported in the literature, such as pulsed electron deposition of CIGS films on the fluorine-doped tin oxide (FTO) or indium tin oxide (ITO)^31^, evaporation of CIGS films on the FTO, ITO, and soda-lime glass (SLG)^33–36^, ITO/SLG, and tin oxide (SnO_2_)^37^, MoO_3_/Mo/SLG, and MoO_3_/ITO/SLG^38^, electrodeposition of CIS films on the flexible ITO/PET substrates^25^, and FTO^39^, and chemical bath deposition of CIS films on glass^40^. There are also limited reports on spray-deposition of CIS films on the polyethylene terephthalate (PET)^41^, borosilicate glass, molybdenum-coated glass, and CdS^42^, spray-deposition of aluminum-doped CIS films on glass, In_2_S_3_/glass, ZnO/glass, and SnO_2_/glass^43^, and spray-deposition of CIS films on top of a single (compact), and a double (compact + porous) ZnO substrates^44^.

In this research, we have used FTO, FTO/nickel oxide (NiO_x_) and FTO/molybdenum oxide (MoO_3_) as substrates to deposit CISe. FTO (Fluorine-doped Tin Oxide) glass is a transparent conductive metal oxide with a work function of −4.9 eV^45^ which is near to the valence band of CISSe (−5.14 eV)^46^. In this regard, to reduce the charge recombination, depositing a hole transporting layer (HTL) in substrate structure or an electron transporting layer (ETL) in the superstrate structure with a high band gap on the FTO glass could be an efficient way. Among different inorganic hole transporting materials, MoO_3_, and NiO_x_ with high hole mobilities, wide bandgaps, and high work functions are the most promising choices to reduce energy barrier for the hole transfer (in the substrate structure) from the valence band of CISe to the FTO^35,38,45–48^. Also, MoO_3_ is a two-dimensional layered structure, chemical activity, easy reproducibility, high transparency (> 80% in the visible and near IR range), wide bandgap (3.0–3.8 eV), and high work function (~ 5.7 eV), and has been utilized in PV and tandem devices^38,49,50^. In addition, NiO_x_ is a wide bandgap (*E*g ~ 3.60 eV) p-type metal oxide by deep valence band around 5.4 eV and suitable work function (*W*_F_) over 5.0 eV, and optimal hole mobility of 0.141 cm^2^ V^−1^ s^−1^, which is usually compact nanocrystalline and is known as one of robust hole transport layers (HTL)^47,51,52^. FTO is one of the most widely used substrates for optoelectronic applications, like liquid crystal displays, organic light-emitting diode displays, touch screens, smart windows, and solar cells^53–55^ due to its wide band gap (> 3 eV), high mechanical hardness, low resistance (< 10^–4^ Ω cm), high optical transparency (> 80%) in the visible region, reasonably low-cost, good stabilities in the acid atmosphere or atmospheric conditions and at high temperature.

This manuscript aims to potentially substitute the molybdenum (Mo) opaque substrate with a transparent substrate in the bifacial photovoltaic device, by using a low-cost, fast, and more industrial method of spray pyrolysis for CISe films. For this purpose, we have investigated the effect of three different substrates (FTO, FTO/NiO_x_, and FTO/MoO_3_) on the optical, structural, morphological, electrochemical, and electrical properties of deposited CISe films by spray pyrolysis.

## Experimental

CuInS_2_ (CIS) and CuInSe_2_ (CISe) thin films were deposited by the chemical spray pyrolysis (CSP) technique from precursor aqueous solutions. Fluorine-doped tin oxide (FTO) conductive glass (15 Ω/sq, Dyesol), FTO/NiO_x_, and FTO/MoO_3_ were used as substrates in this work. Before film deposition, FTO substrates were cleaned for 15 min with detergent, hydrochloric acid (HCl), acetone, and ethanol, respectively in an ultrasonic bath and then heat-cleaned for 30 min at 500 °C.

### Material types

The CIS precursor solutions were prepared using a mixture of copper iodide (CuI, Merck, 98%), Indium (III) chloride (InCl_3_, Strem Chemicals, 99.99%), and thiourea (CH_4_N_2_S, Acros, 99%) salts as copper, indium, and sulfur sources, respectively, by dissolving in de-ionized water (DI-water). The concentration of copper, indium and sulfur was 0.08 M, 0.1 M, and 0.4 M, respectively, which was held constant for all experiments. All chemicals were used without further purification. Molybdenum (VI) oxide (MoO_3_, Sigma-Aldrich, ACS reagent, ≥ 99.5%) was used as a molybdenum (VI) oxide source. Nickel (II) acetate tetrahydrate (98%), methanol (99.8%), and diethanolamine (≥ 98.0%) were purchased from Sigma-Aldrich which were used as nickel and solvent sources.

### Deposition method

#### Molybdenum oxide (MoO_3_)

We deposited MoO_3_ thin films in this research. First, the FTO substrates were placed into a DC magnetron sputtering system to deposit a stack of MoO_3_ films on the rear side. Deposition time is 15 min with a power of 150 W. MoO_3_ films deposition were done by thermal evaporation method in a chamber with a base pressure of approximately 2 × 10^−5^ Torr. The resulting MoO_3_ films were then annealed on a hot plate, in an air environment, at 150 °C temperature for 5 min to generate the oxygen vacancy defects.

#### Nickel oxide (NiO_x_)

NiOx was prepared by a sol–gel method. The sol–gel mixture was prepared by dissolving nickel (II) acetate tetrahydrate in methanol at a concentration of 0.3 M. Then an equimolar amount of diethanolamine was added and the mixture was stirred and heated on a hotplate at 70 °C. The NiO_x_ films were deposited by spin coating at 2000 rpm for 30 s on FTO substrates at room temperature. This process was repeated three times. The NiO_x_ films were then annealed at 350 °C for 1 h in ambient air^47^.

#### Copper indium selenide (CISe)

**Figure 1:**
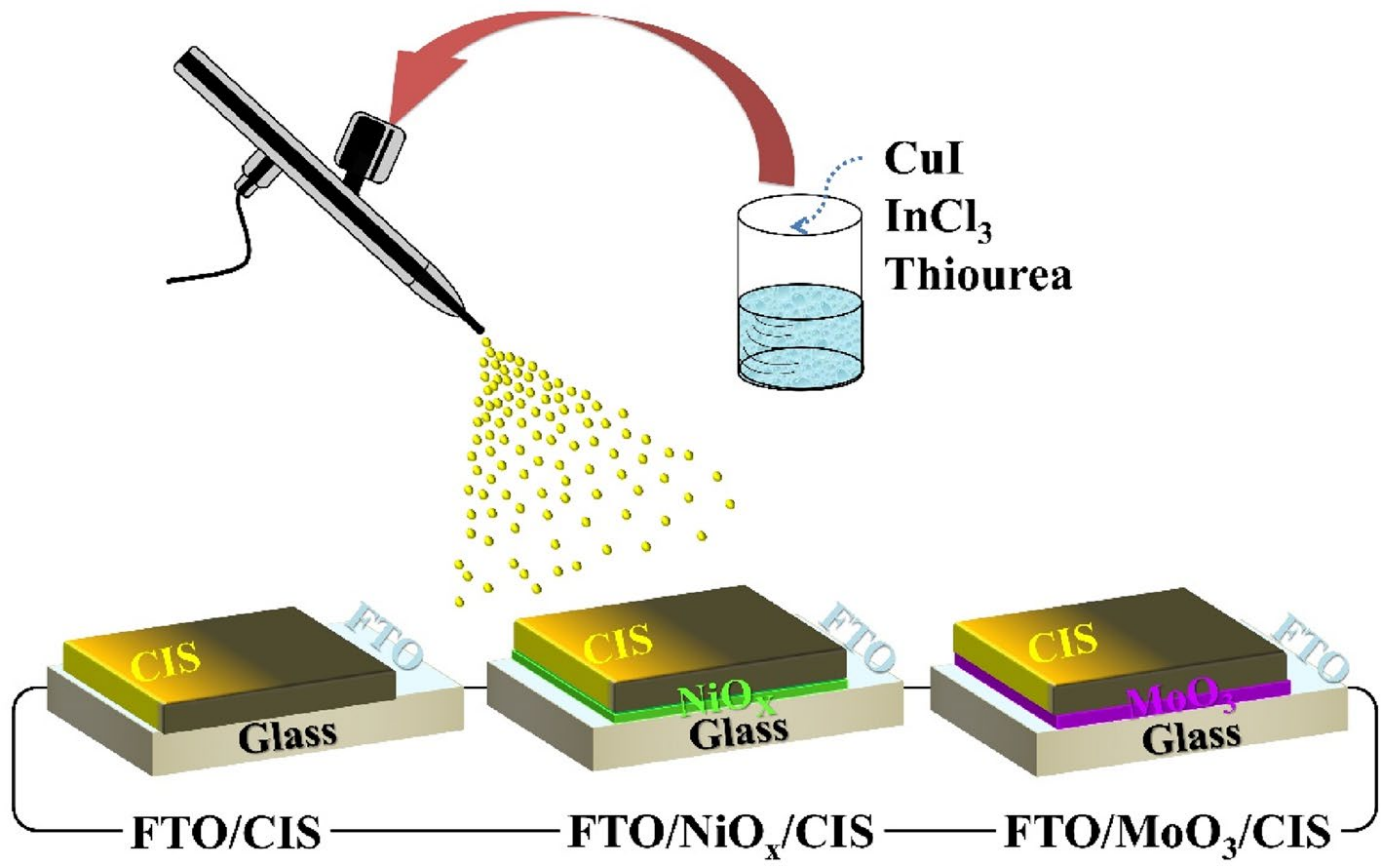
Schematic presentation of the spray deposition method.

FTO, FTO/NiO_x_ and, FTO/MoO_3_ were used as substrates. Based on initial studies, some parameters were assumed to be constant in this research such as rate of deposition: 4 ml/min, the distance between hot plate and nozzle: 15 cm, and deposition temperature: 350 °C. These parameters were elected to avoid wet droplet stains on the substrate at low temperature/high deposition rate conditions, also powdery film formation at high temperature/low deposition rate conditions. The desired deposition regime lies between these two extremes. The solution was sprayed by air as carrier gas and using a conventional airbrush. Conditions during selenization have an extreme influence on crystallization and large grain formation. The selenization process was done for ten 1.4 × 1.4 cm^2^ films in a graphite box with elemental Se pellets. Before starting the selenization process, the furnace tube was first purged with nitrogen gas and then was set at a predetermined pressure of 500 Torr. The heating profile lasted for 40 min, containing ramping up (~ 25°C min^−1^) to 500 °C for 20 min and was resided for 20 min. For ease of reference, the films formed on different substrates are named as *FTO:*(FTO/CISe) film, *FTO/NiO*_*x*_*:* (FTO/NiO_x_/CISe) film, *FTO/MoO*_*3*_*:* (FTO/MoO_3_/CISe) film. A schematic of film deposition by the spray method can be seen in Fig. 1.

### Characterization

The morphology, composition and crystal structure of different CIS(e) films before and after selenization were examined by high-resolution field emission scanning electron microscopy (FESEM; HRSEM, XL30SFEG Phillips Co., Holland at 10 kV), energy dispersive spectroscopy (EDS; EDAX Genesis apex, acceleration voltage: 30 kV). To measure the roughness, atomic force microscopy (AFM) (VEECO-CP research) was used with a silicon tip of 10 nm radius in tapping mode. The crystal structure of the as-sprayed thin films was analyzed by X-ray diffraction (XRD) technique (X’Pert Pro MPD, PANalytical) with CuKα (λ = 1.5406 Å) radiation in the 2*θ* range from 4° to 80°. The scanning mode is continuous with a step size of 0.02°and scan step time of 0.5 s. The optical properties of the deposited layers were evaluated by measuring the transmittance spectra by Ultraviolet–Visible (UV/Vis) spectroscopy (Lamda 25, Perkin Elmer). The Mott-Schottky (MS) analysis was performed in a three-electrode system, in 0.5 M Na_2_SO_4_ solution (pH 6.0) as an electrolyte using an EIS-26H system (IRASOL). The working, reference and counter electrodes were (*FTO*, *FTO/NiO*_*x*_, *FTO/MoO*_*3*_), Ag/AgCl (3 M KCl), and Pt rod, respectively. The frequency of the signal was 1 kHz, and the bias voltage was scanned from − 0.8 V to 0.3 V, with 50 mV s^−1^ speed (peak-to-peak) at ambient conditions. All experiments proceeded after 5 s electrode stabilization. Charge mobility of CISe films was measured using Keithley 2400 Source meter.

## Results and discussion

### Morphological and structural properties

**Figure 2:**
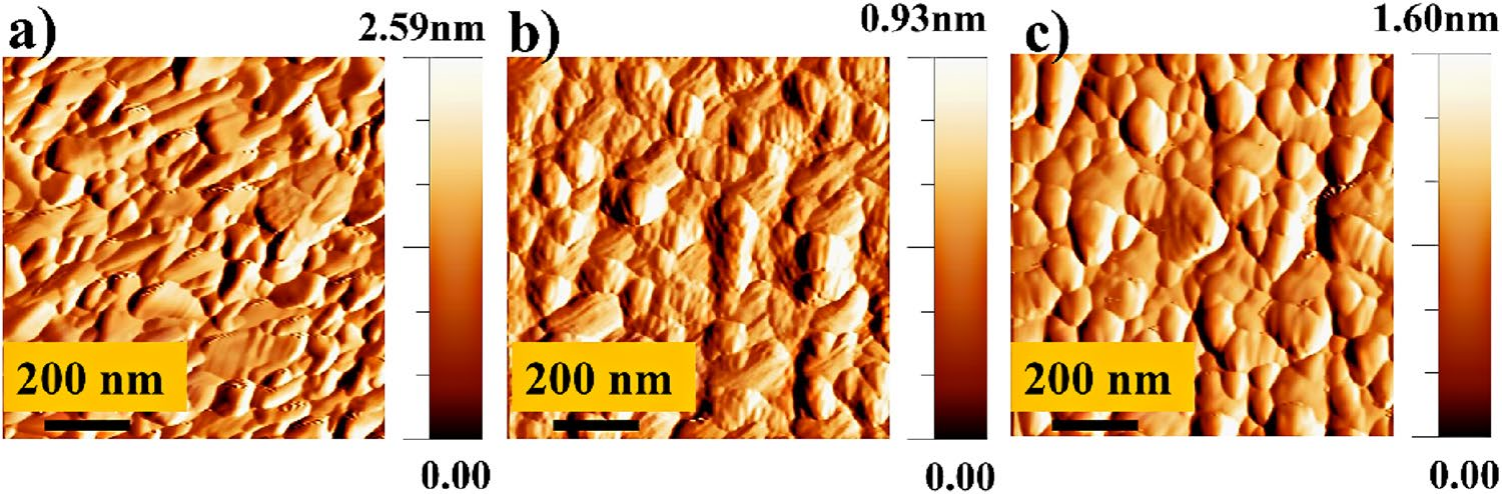
AFM images of various substrates of (**a**) FTO, (**b**) FTO/NiO_x_, and (**c**) FTO/MoO_3_.

The AFM topographical images of different substrates including FTO, FTO/NiO_x_, and FTO/MoO_3_ are shown in Fig. 2. According to AFM images, the surface roughness of FTO is 16.5 nm. After deposition of the NiO_x_ and MoO_3_ layers, the surface roughness decreases to 10.9 and 12.8 nm, respectively. Similar roughness reductions were previously reported for FTO, FTO/SnO_2_, and FTO/SnO_2_/CdS surfaces in which the surface roughness decreases by deposition of CdS nanoparticles^56^.

According to the results of the FESEM cross-sectional images, the thickness of the NiO_x_ and MoO_3_ layers deposited onto the FTO are 28 and 36 nm, respectively, Fig. S1.

**Figure 3:**
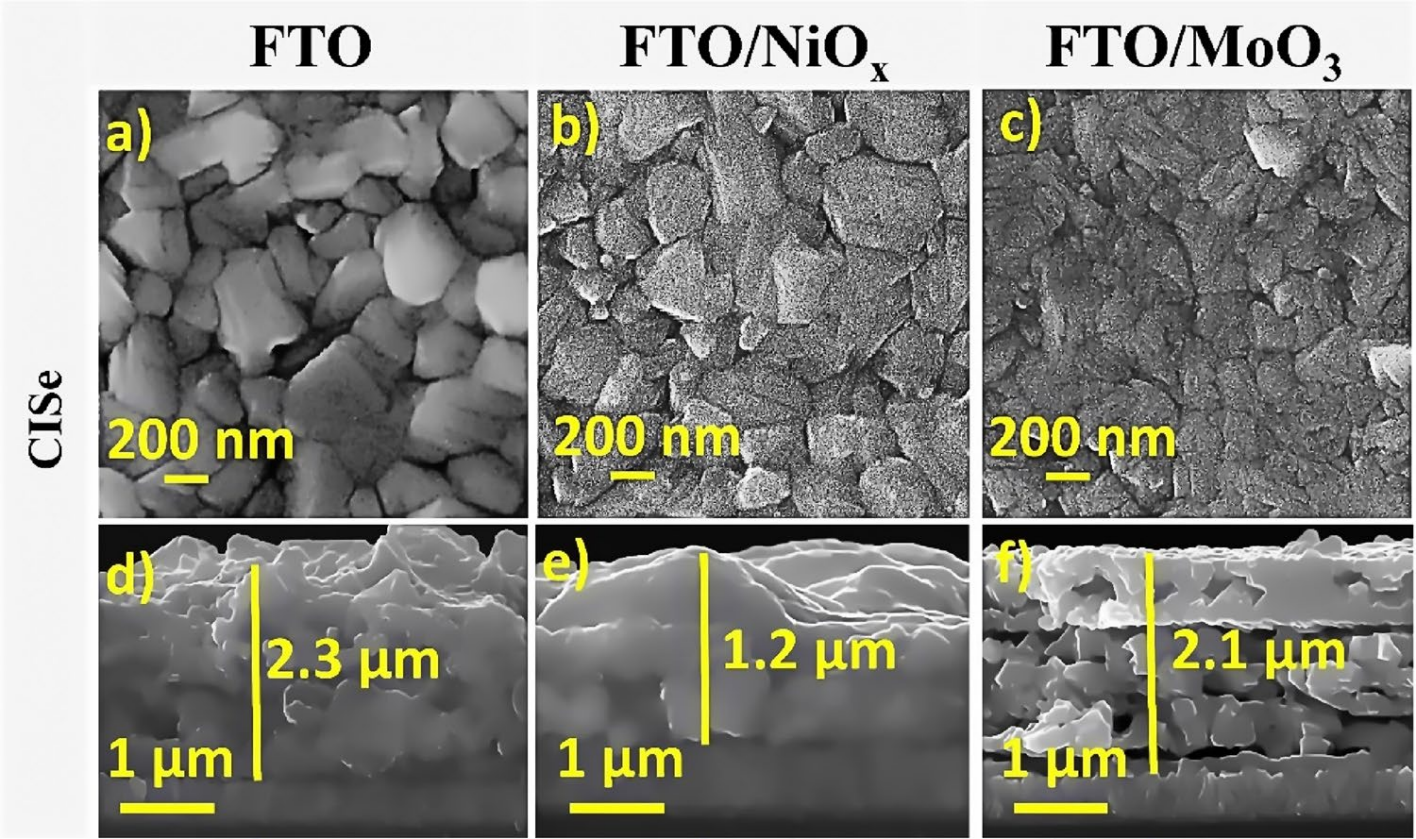
Surface and cross-sectional FESEM images of CISe films on: (**a,d**) FTO, (**b,e**) FTO/NiO_x_, (**c,f**) FTO/MoO_3_.

Figure 3 represents the FESEM surface and cross-sectional images of CISe thin films on different substrates. As shown in FESEM surface images (Fig. 3a–c), all deposited films show dense and crack-free surface morphology while the *FTO* films show larger grains compared to other films (Fig. 3a). The largest grain size was calculated using ImageJ software which was estimated to be ~ 0.50 μm (Table 1). The FESEM surface images of CISe films are also shown in smaller magnifications in Supporting Information, Fig. S2.

**Table 1:** The grain size of CISe films deposited on different substrates.

	FTO	FTO/NiO_x_	FTO/MoO_3_
CISe grain size (µm)	0.50	0.42	0.15

Cross-sectional FESEM images of CISe films on various substrates have been shown in Fig. 3d–f. The morphology of deposited CISe films on the FTO substrate looks very similar to the case of CISe growth on FTO/NiO_x_ substrate, hence the thickness of the film appears to be affected by the type of substrate. The deposited CISe films on the FTO substrate have the highest roughness substrate and film thickness is about 2.3 µm (Fig. 3b), while CISe deposition on *FTO/NiO*_*x*_ substrate leads to the lowest roughness substrate and thickness of 1.2 µm (Fig. 3e). On the other hand, the CISe films grown on FTO/MoO_3_ substrate show a bilayer structure in which the small and large grains are placed at the bottom and the top surface, respectively. As shown in Fig. 3f pores in the film can act as pathways for the evaporation of volatile materials (such as Cl_2_ or In_2_Se) during the selenization process^57^. Similar results were previously reported for doctor blade coating of CIS precursor solutions on Mo substrates^58^, solution-processing of amorphous nanoparticle-based CISe films on the Mo substrate^59,60^, which selenization of the CIS films with Se vapor at high-temperature results in bi-layered films with an upper layer of chalcopyrite CISe and a small grain-sized bottom layer.

The exact influence of this bilayer on different film properties and the resulting device is not still clear, and contradictory views are present. Generally, the generated bi-layered CISe films have been attributed to the presence of carbon at the bottom^58–60^, formation of CIS on top of the layer and hinder further evaporation of solvent^58^, the existence of mixed-phases, consisting of the ordered vacancy compound (OVC), Cu–Se phases, CISe and trace of CIS in the top layers^59^. In this study, the probable cause for creating a bilayer structure for CISe film growth on FTO/MoO_3_ substrate is the existence of impurity.

The composition of each sample was determined by EDS analysis (see Fig. S3 and Table S1). The compositional ratio of CISe films deposited on FTO, FTO/NiO_x_, and FTO/MoO_3_ substrates showed that the S/In ratio was variable from about 0.37 to 0.53. Thus, the films are described as CuIn(S_y_Se_1−y_)_2_ because of the presence of ~ 9 to 14% atomic sulfur determined using EDS. The elemental compositions demonstrate that the atomic percentage (48.8 at.%) of selenium is higher for the CISe film on the FTO substrate than those deposited on other substrates, which proposes lower selenium vacancies.

Also, the CISe films have a Cu/In ratio in the range of 0.77–1.04. The atomic ratio of Cu/In in the CISe film on the FTO substrate is larger than that of the initial solution before depositing. These results revealed that the films changed from the In-rich CISe phase, in the *FTO/NiO*_*x*_ and *FTO/MoO*_*3*_ films to the stoichiometric CISe phase. This phenomenon can be described by the formation of volatile In_2_Se during the selenization process and subsequent evaporation due to the high vapor pressure of In_2_Se^61,62^. As confirmed by EDS results, there is a minor Cl residue (0.08 at%) in the *FTO* films and a major chlorine (Cl) residue (0.50 at%) in the *FTO/MoO*_*3*_ films within the EDS resolution. The high chlorine residual content indicates an incomplete reaction between the precursor ingredients in the *FTO/MoO*_*3*_ films and can lead to the formation of a bilayer structure in these films. These results are in good agreement with the results of the FESEM-analysis.

**Figure 4:**
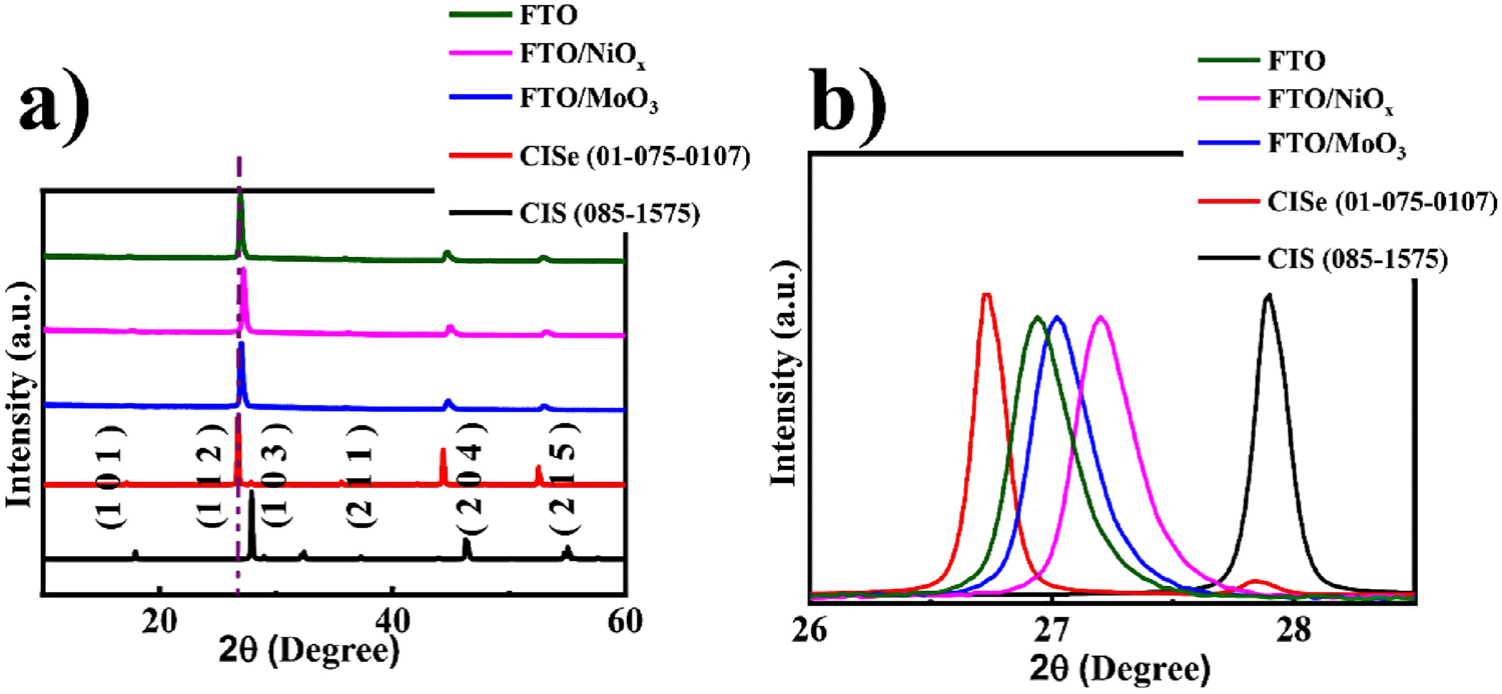
XRD pattern of CISe films on different substrates.

Figure 4 represents the XRD patterns obtained from the CuInSe_2_ films used for structural and materials identification study. It can be observed that three prominent peaks identified as the planes of (112), (204), and (215) corresponding to CuInSe_2_ chalcopyrite tetragonal crystal structure and are in good agreement with the standard JCPDS file (standard JCPDS no. 085-1575 and 01-075-0107) for CIS and CISe films, respectively. In addition, no peaks of other impurities such as Cu_x_Se, In_2_S_3_, etc. were detected, indicating the high phase purity of CuInSe_2_ films (Fig. 4a). However, considering the used CISe reference card, a noticeable peak shift towards higher diffraction angles can be observed considering the reflections of the detected chalcopyrite phase. This peak shift can be described by a non-complete substitution of sulfur with selenium. The smaller atomic radius of the remaining sulfur compared to selenium results in the smaller unit cell of the chalcopyrite phase, which causes a peak shift towards higher diffraction angles and forms the CI(S, Se) alloy (Fig. 4b)^63^.

Figure S4 demonstrates the XRD pattern obtained from the NiO_x_ and MoO_3_ films compared to standard prominent peaks identified as the planes of (1 1 1), (2 0 0), and (2 2 0) demonstrating a cubic crystal structure for NiO_x_ thin films and planes of (0 2 0), (1 2 1) and (1 5 0) showing an orthorhombic crystal structure for MoO_3_which is in good agreement with the standard JCPDS file (standard JCPDS no. 00-002-0422 and 01-076-1003) for NiO_x_ and MoO_3_ films, respectively.

To research, the structural properties of CISe films such as dislocation densities, micro-strain, the number of crystallites per unit area, etc. have been calculated from the major (112) peak of X-ray microbeam studies. The dislocation density (*δ*) presents information about the crystal structure of CISe films, which can be evaluated using Williamson and Smallman’s equation (Eq. 1)^64^:

1 $$\delta = { 1}/D^{{2}}$$

where *D* is the crystallite size.

The micro-strain (*ε*) influences the optoelectronic properties of the CISe thin films due to the distorted lattice. The average micro-strain present in the CISe films was calculated by Eq. (2)^65^:

2 $$\varepsilon = \beta cos\theta /{4}$$

where *β* is the full peak width at half maximum (FWHM)and *θ* is the Bragg angle.

Furthermore, the number of crystallites per unit area (*N*) was estimated by using the following equation^66^:

3 $$N = t/D^{{3}}$$

where (*t*) is the thickness of the film.

As reported in Table 2, the dislocation density of films is found to increase from 4.52 to 44.44 [(lines m^−2^) × 10^−2^] with changes in the substrate from FTO to FTO/MoO_3_. This indicates that the crystallinity of the films impairs for FTO/MoO_3_ substrate^67^. Hence, the suitable structural properties in terms of good crystallinity and lowest dislocation density belong to the FTO substrate. On the other hand, values of micro-strain decrease from 5.12% for FTO/MoO_3_ substrate to 4.52% for FTO substrate. This is ascribed to the decrease of defect level and grain boundaries due to improved crystallinity and increased grain size for FTO substrate.

**Table 2:** Structural parameters of the CISe films deposited on various substrates.

Substrate type	Bragg angle (°)	FWHM (°)	*δ* (lines cm^−2^) × 10^8^	ε × 10^−2^	N × 10^8^ (cm^−2^)
FTO	26.92	0.20	4.52	4.45	22.15
FTO/NiO_x_	27.04	0.21	6.25	4.67	18.75
FTO/MoO_3_	27.24	0.23	44.44	5.12	622

The minimum amount of dislocation density and the micro-strain of the CISe films were 4.52 × 10^14^ and 4.45 × 10^–2^ cm^−2^, respectively. These values are significantly lower than the spray pyrolyzed CuInGaS_2_ and CuAlS_2_ films in literature^68,69^. The reduction in the dislocation density and micro-strain for FTO substrate was most probably due to the stress relaxation, which occurs during the recrystallization process^69^.

Also, the number of crystallites per unit area significantly changes with the substrate type. The number of grains increased notably up to about 622 × 10^8^ cm^−2^ for FTO/MoO_3_ substrate, (Table 2). According to Table 1, the results show that the type substrate has an important effect on the grain size of the CISe films. The grain size increases to less than 0.47 µm for the FTO substrate and decreases to 0.15 µm for the FTO/MoO_3_ substrate.

This result is probably due to the highly increased growth rate in FTO/MoO_3_ substrate, which results in lower grain size and a higher number of crystallites per unit area in the films^68,69^.

### Optical properties

**Figure 5:**
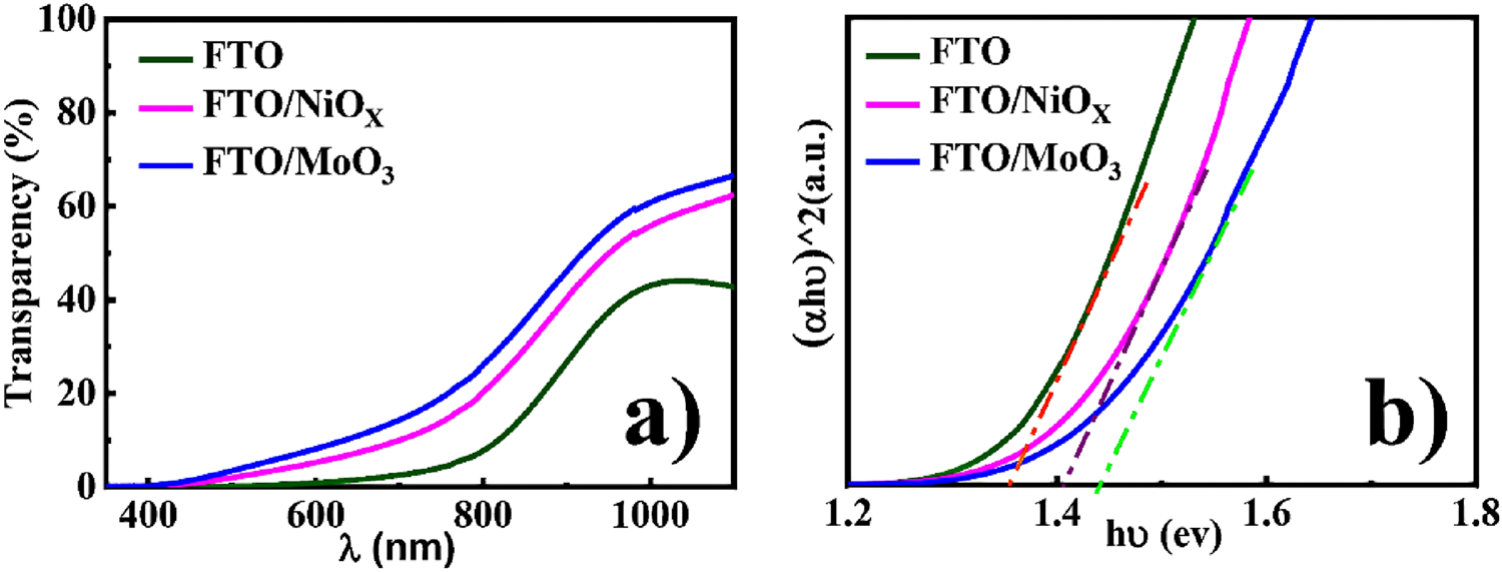
(**a**) Optical transmittance spectra, (**b**) direct band gap (eV) of CISe films deposited on different substrates measured by UV–Vis analysis.

The optical transmittance spectra of CISe films by various substrates within the range of 350–1100 nm are shown in Fig. 5a. UV–Vis transmittance spectra can be used to extract the band gap energy of the films using the Tauc plot formalism^70^:

4 $$\mathrm{\alpha }\cong \frac{{A\left(h\nu -{E}_{g}\right)}^{n}}{h\nu }$$

where *A* is a constant, n = 0.5 for allowed direct band transition, *h* is the Planck constant, *α* is the absorption coefficient near the absorption edge and *E*_*g*_ is the optical band gap value. The optical bandgap energy of the CISe films has shown in Fig. 5b.

Moreover, the optical transmittance slightly decreased with depositing CISe films on FTO. The transmittance of this film was approximately 40%. Figure 5a shows the absorption edge shifts to shorter wavelengths with the variation of the substrate type from *FTO* to *FTO/NiO*_*x*_ and *FTO/MoO*_*3*_ for films. From the Tauc plot analysis (Fig. 5b), energy band gap values of 1.35, 1.41, and 1.44 eV were found for *FTO*, *FTO/NiO*_*x*_*, *and *FTO/MoO*_*3*_ CISe films, respectively (Table 3).

**Table 3:** The direct band gap (eV) of CISe films deposited on different substrates was calculated by UV-Vis analysis.

Substrate	*FTO*	*FTO/NiO*_*x*_	*FTO/MoO*_*3*_
Direct band gap (eV)	1.35	1.41	1.44

The values of the band gap are slightly larger than the previously reported solution-based CISe thin films, with values of 1.00 eV^71^, 1.04^72^, 1.06 eV^73^, and 1.15 eV^74^, which may be due to the small amount of residual sulfur in the CISe films^71^, as shown by the EDS and XRD results. Also, a further decrease in band gap value for *FTO* films may be due to crystallinity improvement^75–77^. The band gaps are higher for *FTO/NiO*_*x*_ and *FTO/MoO*_*3*_ films with smaller crystalline sizes, which may be due to the density of states at the interfaces, grain boundaries, and the defects energy level on the surface^78,79^.

## Electrochemical properties

**Figure 6:**
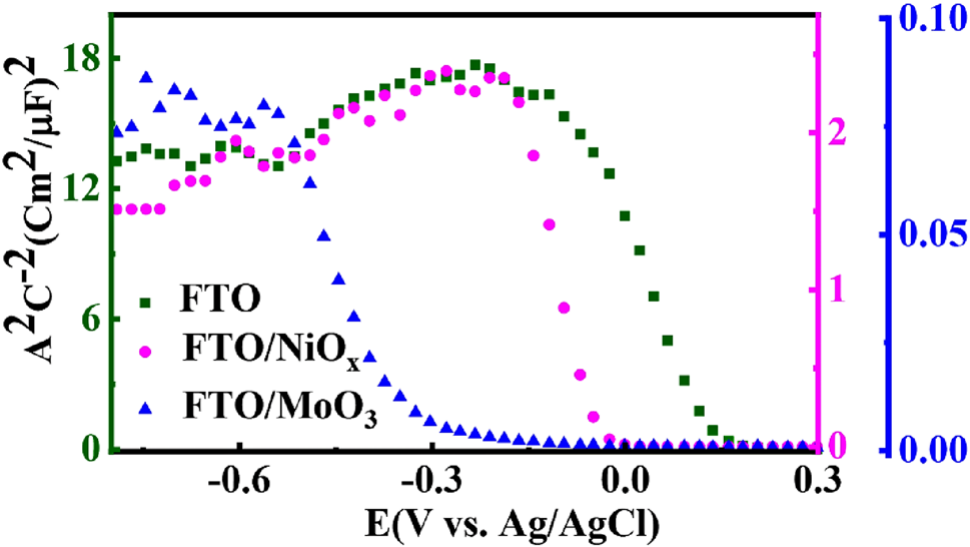
Mott–Schottky plot of CISe films deposited on different substrates measured in 0.5 M Na_2_SO_4_ solution.

To further investigate the effect of the substrate on the electrochemical properties of CISe films, we have measured the Mott Schottky (M–S) relationship based on the capacitance versus applied potential. The Mott Schottky equation is given as follows^80^:

5 $$\frac{{A}^{2}}{{\mathrm{C}}_{\mathrm{SC}}^{2}}=\frac{2}{{\upvarepsilon }_{0}{\upvarepsilon }_{\mathrm{r }}{\mathrm{eN}}_{\mathrm{A}}}(\mathrm{V}-{\mathrm{V}}_{\mathrm{fb}}-\frac{\mathrm{KT}}{\mathrm{e}} )$$

where *C*_sc_ is the space charge capacitance, *ε*_r_ is the dielectric constant of the CISe film (13.6)^81^, *ε*_0_ is the permittivity of a vacuum, *e* is the electron charge, *A* is the film surface area in contact with the electrolyte, *N*_A_ is the density of acceptor in the semiconductor, *V* is the externally applied potential, *V*_fb_ is the flat band potential, *k* the Boltzmann constant (1.38 × 10^−23^ J K^−1^) and *T* the operation temperature (300 K). The negative slope in Fig. 6 (M–S plots) indicates that all CISe thin films are p-type semiconductors.

The carrier density (*N*_A_) can be also conveniently found by determining the slope of the linear region of the M–S plot by using Eq. (5)^77^. The semiconductor parameters such as values of the *V*_fb_, the carrier density *N*_A_ width of the space-charge region (SCR), *W*, and energy level have been shown (Table 4).

**Table 4:** Information on Mott-Schottky analysis for CISe films deposited on a different substrate.

Substrate	*FTO*					*FTO/NiO*_*x*_					*FTO/MoO*_*3*_				
Energy (eV)	*V*_fb_	*E*_c_	*E*_f_	*E*_v_	*N*_A_ (cm^−3^)	*V*_fb_	*E*_c_	*E*_f_	*E*_v_	*N*_A_ (cm^−3^)	*V*_fb_	*E*_c_	*E*_f_	*E*_v_	*N*_A_ (cm^−3^)
	0.13	−3.67	−4.84	−5.02	1.6 × 10^17^	−0.07	−3.22	−4.63	−4.64	5.4 × 10^17^	−0.37	−2.90	−4.33	−4.34	2.4 × 10^19^
W (nm)	34.94					13.96					4.81				

*N*_A_ is estimated as 1.6 × 10^17^, 5.4 × 10^17^, 2.4 × 10^19^ cm^−3^ with a variation of the substrate type from *FTO* to *FTO/NiO*_*x*_ and *FTO/MoO*_*3*_ films, respectively. These results indicate that the carrier density concentration in the CISe absorber does not change significantly by the vary of substrate type from *FTO* to *FTO/NiO*_*x*_ films.

Although the carrier density values of *FTO* and *FTO/NiO*_*x*_ films are close to previous data reported by the solution method^82–87^, carrier density values of *FTO/MoO*_*3*_ films are nearly high compared to the vacuum-based deposition method^85,88,89^. In this work, the high values of carrier density for *FTO/MoO*_*3*_ films could be due to the presence of more grain borders and grain boundaries^85^, roughness and non-planar interfaces on the surface^85,90^, impurities like Cl^91^, as evidenced by the FESEM and EDS analysis.

An important parameter in solar cells or other electronic devices is *V*_fb_, which controls the band alignments and carrier transfer at the interfaces^92^. The flat-band potential of the semiconductor can be calculated by intersecting the *V*-axis of the linear region of the M–S plot^77^. The *V*_fb_ value shifts significantly from 0.13 to −0.37 V (vs. Ag/AgCl) with a variation of the substrate type from *FTO* to *FTO/NiO*_*x*_ and *FTO/MoO*_*3*_ films. This shift can be related to the change in the morphology and the composition of elements by changing the substrate type^77^. The *V*_fb_ value for CISe films with *FTO* substrate is more positive than those obtained by other films, that is indicating the better conductivity of *FTO* thin films due to an increase in their crystallinity^93^ which is confirmed by FESEM data.

The width of the SCR, *W*, is directly related to the capacitance of the CISe films. Equation (6) gives^94^:

6 $$W=\sqrt{\frac{2\varepsilon {\varepsilon }_{0}{V}_{fb}}{q{N}_{A}}}$$

Using this equation, the width of the space-charge layer results in a maximum value of about 34.9 nm for CISe films with *FTO* substrate. The broader space-charge region in *FTO* films can increase the accumulation of charge carriers^80^. Utilizing the other substrate seems quite to reduce the SCR width in the CISe films (to about 4.81 nm), which leads to the limited short-circuit current density (*J*_sc_) values obtained in solar cells^95^. This would prove that the electrically active region of the CISe films is different for the various substrates.

### Electrical properties

**Figure 7:**
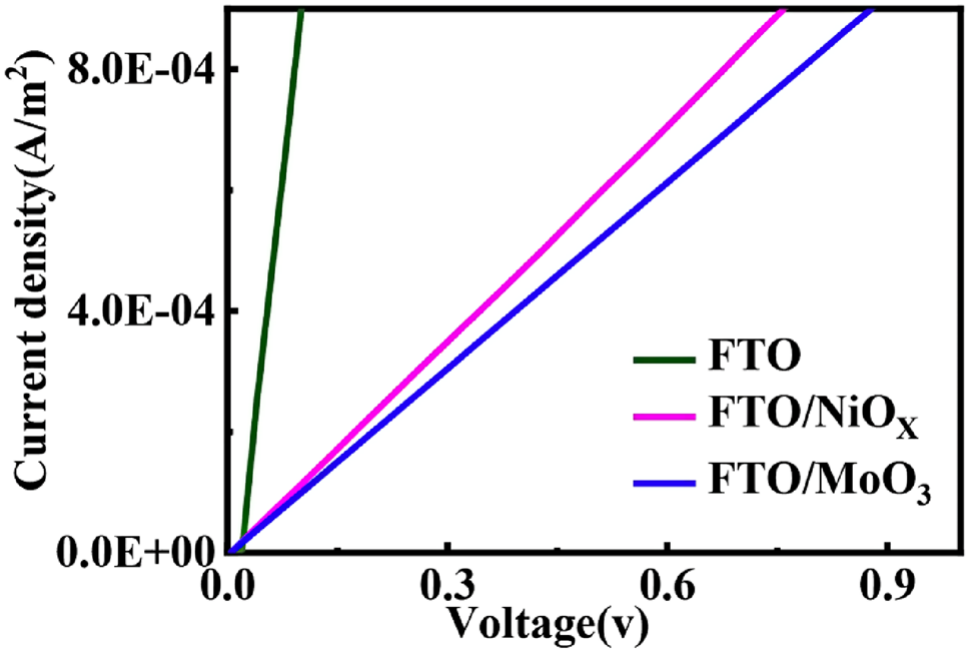
Dark *J–V* analysis for films CISe on different substrates.

To understand more deeply the effect of the substrate type on the hole mobility, conductivity, bulk electrical resistivity, diffusion coefficient, and electrical behavior in the absorber layers, the current density–voltage (*J–V*) characteristics were recorded in the dark conditions and at ambient temperature. Figure 7 describes the typical curves of current density (*J*) as a function of the applied potential (*V*) for *FTO*, *FTO/NiO*_*x*_*, *and *FTO/MoO*_*3*_ films. For all films, the dark *J-V* analysis indicates a linear characteristic that means good ohmic contacts without an intermediate layer of Mo between CISe films and substrate.

The devices were fabricated with the structure of FTO/CISe/Graphite. Then, the following equation is used to calculate the mobility in the ohmic region^96^:

7 $$J \, = \, N_{A} e\mu V/d$$

where *J* is the current density, *N*_A_ is the carrier density, *e* is the electronic charge, *μ* is the hole mobility, *V* is the applied voltage and *d* is the distance between the electrodes, (the thickness of the thin film). The electrical parameters of all corresponding films were summarized in Table 5.

**Table 5:** Electrical parameters of the CISe films with various substrates.

Substrate type	µ (cm^2^ V^−1^ s^−1^)	σ (S m^−1^)	ρ (Ω cm)	D (cm^2^ s^−1^)
FTO	7.37 × 10^–2^	1.88 × 10^–3^	0.53 × 10^3^	1.91 × 10^–3^
FTO/NiO_x_	2.08 × 10^–2^	1.78 × 10^–3^	0.56 × 10^3^	5.38 × 10^–4^
FTO/MoO_3_	1.17 × 10^–3^	4.49 × 10^–3^	0.22 × 10^3^	3.03 × 10^–5^

Hole mobility values of 7.37 × 10^–2^, 2.08 × 10^–2^, and 1.17 × 10^−3^cm^2^ V^−1^ s^−1^were obtained for *FTO*, *FTO/NiO*_*x*_*, *and *FTO/MoO*_*3*_ films, respectively. The measured hole mobility values for *FTO* and *FTO/NiO*_*x*_ films are about one order of magnitude larger than *FTO/MoO*_*3*_ film, which indicates better crystallinity, uniformity, and grain boundary continuities in these thin films. On the other hand, the larger grain size in *FTO* and *FTO/NiO*_*x*_ films is desirable as it leads to less grain boundary scattering of the charge carriers, i.e., better electrical transport properties^97^. Although the hole mobility values for *FTO* and *FTO/NiO*_*x*_ films are slightly lower than the previously reported data^88,98^. The low value of mobility in *FTO/MoO*_*3*_ films may be attributed to impurities (i.e. 0.50 atomic% chlorine), which can act as dopants or cause traps that increase recombination or reduce mobility. Moreover, because *FTO/MoO*_*3*_ films have poor crystallinity and more grain boundary scattering, grain boundary discontinuities and presence of surface states^99^ specific surface area and a border effect may intensify carrier scattering at the surface and reduce mobility^85^.

Both the carrier density and the hole mobility contribute to the bulk electrical resistivity and conductivity. The conductivity of the CISe thin films is proportional to the carrier density and hole mobility^100^:

8 $$\sigma \, = \, eN_{A} \mu$$

where *σ* is conductivity, e is the electronic charge, *N*_A_ is carrier density and *µ* is hole mobility. The bulk electrical resistivity values were calculated using the following well-known equation^101^:

9 $$\rho =\frac{1}{\sigma }$$

where *ρ* is bulk electrical resistivity and *σ* is conductivity.

Table 5 shows the substrate type dependence of the bulk resistivity and conductivity of CISe films. The bulk resistivity of the CISe films was about 5.6 × 10^2^ to 2.2 × 10^2^ Ωcm. These values are similar to the reported values which are in the range 4.3 × 10^2^–5.3 × 10^2^ Ωcm^102,103^. Also, the conductivity of all films was between 1.78 × 10^–3^ to 4.49 × 10^–3^ S cm^−1^.

The charge carrier diffusion length in a semiconductor is described by the average distance that charge carriers travel in a semiconductor. The diffusion coefficient and mobility of charge carriers are related by Einstein's equation^104^:

10 $$D=\mu \frac{KT}{e}$$

where *D* is diffusion coefficient, *µ* is hole mobility, *k* is the Boltzmann constant (1.38 × 10^−23^ J K^−1^), *T* is the operation temperature (300 K) and *e* is the electronic charge.

The CISe films prepared using various substrates show hole diffusion coefficients from 10^–3^ to 10^–5^ cm^2^ s^−1^. The *J–V* dark measurements results reveal that the hole diffusion coefficient of the *FTO* films is 1.91 × 10^−3^ cm^2^ s^−1^, which is higher than the *FTO/NiO*_*x*_ (5.38 × 10^−4^ cm^2^ s^−1^) and *FTO/MoO*_*3*_ (3.03 × 10^−5^ cm^2^ s^−1^) films. The higher hole diffusion coefficient value is favorable for fast charge transport and results from the effective connection of the grains to create the charge carrier's continuous pathway in the CISe films^105^. However, the existence of deep levels in the CISe films is unsuitable since they act as recombination centers for charge carriers and therefore reduce carrier diffusion coefficients in *FTO/NiO*_*x*_ and *FTO/MoO*_*3*_ films^103^.

In addition, the diffusion coefficients calculated from the other research have been reported 10^−16^ cm^2^ s^−1^ and 10^−7^ cm^2^ s^−1^ for CuIn(Se,S)_2_ crystals^106,107^, 10^−14^ cm^2^ s^−1^ for CuInSe_2_ quantum dots^108^, 10^−6^ cm^2^ s^−1^ for Cu(In,Ga)Se_2_ thin films^109^, which are notably lower than the estimated values in this work.

## Conclusion

In this research, transparent substrates for the replacement of molybdenum (Mo) opaque substrate were studied for use in bifacial photovoltaic devices. Three transparent substrates (FTO, FTO/NiO_x_, and FTO/MoO_3_) were used as substrates, and CISe thin films were deposited by spray pyrolysis and selenization. The results of different characterization techniques have a good correlation to each other. The optical transmittance and significant band gap energy of CISe films were changed from 1.35 to 1.44 eV depending on the substrate type. The CISe films deposited on the FTO substrate were more compact, thicker, with larger grains than others. The XRD peaks confirm that all films show a chalcopyrite tetragonal structure without any impurity phase but structural parameters such as micro-strain(ε) of ~ 4.45 × 10^–2^, number of crystallites per unit area (*N*) of ~ 22.15 × 10^8^ cm^−2^ and dislocation density (*δ*) of ~ 4.52 (lines cm^−2^) × 10^8^ have the lowest values for CISe films on FTO substrates. All CISe films are p-type semiconductors with a carrier density of ~ 10^17^ to 10^19^ cm^−3^. Flat band potential and space-charge layer values of the CISe films are estimated based on the Mott-Schottky analysis to be 0.13 V (vs. Ag/AgCl) and 34.94 nm for FTO substrates, respectively. Dark *J–V* measurement exhibited that the CISe films have ohmic behavior with a favorable hole mobility of around 7.37 × 10^−2^ cm^2^ V^−1^ s^−1^ and diffusion coefficient of 1.91 × 10^−3^ cm^2^ s^−1^ for CISe film deposited on FTO substrate which is notably higher than the other two films. Generally, the optical, physical, and electrical properties of CISe films are influenced by substrate type. It is thought that the properties of CISe thin films deposited on the FTO substrates are considerably close to the properties essential for photovoltaic applications, thus FTO can be an alternative substrate to opaque substrates with deposited CISe films as absorber layer, hole transport layer, and photoanode used in applications such as bifacial and tandem solar cell, supercapacitor and sensor.

## Supplementary Information

Supplementary Information.

## Data Availability

All data generated or analysed during this study are included in this published article [and its supplementary information files].
