# Supplementary material for: Effect of transparent substrate on properties of CuInSe2 thin films prepared by chemical spray pyrolysis
Source: Sci Rep. 2022 Aug 30;12:14715. doi: 10.1038/s41598-022-18579-w (PMC9427856; doi:10.1038/s41598-022-18579-w)
Supplement: Supplementary file 1 — Supplementary Information. [file 41598_2022_18579_MOESM1_ESM.docx]

**Supporting Information**

**Effect of** **transparent substrate on properties of CuInSe_2_ thin films prepared by chemical spray pyrolysis**

**Maryam Hashemi^a^, Zahra Saki^b^, Mehdi Dehghani^b^, Seyed Mohammad Bagher Ghorashi^a*^,Fariba Tajabadi^c*^ ,Nima Taghavinia^b^***

*^a^ Department of Laser and Photonics, University of Kashan, Kashan, Iran*, *POBOX 873175-3153*.

*^b^ Department of Physics, Sharif University of Technology,* *Tehran, Iran* *POBOX 11155-9161.*

*^c^* *Department of Nanotechnology and Advanced Materials, Materials and Energy Research Center, Karaj, Iran, POBOX 31787-316.*

Figure S1: FESEM Cross micrograph of various substrates; *a)* FTO/NiO_x_, *b)* FTO/MoO_3_

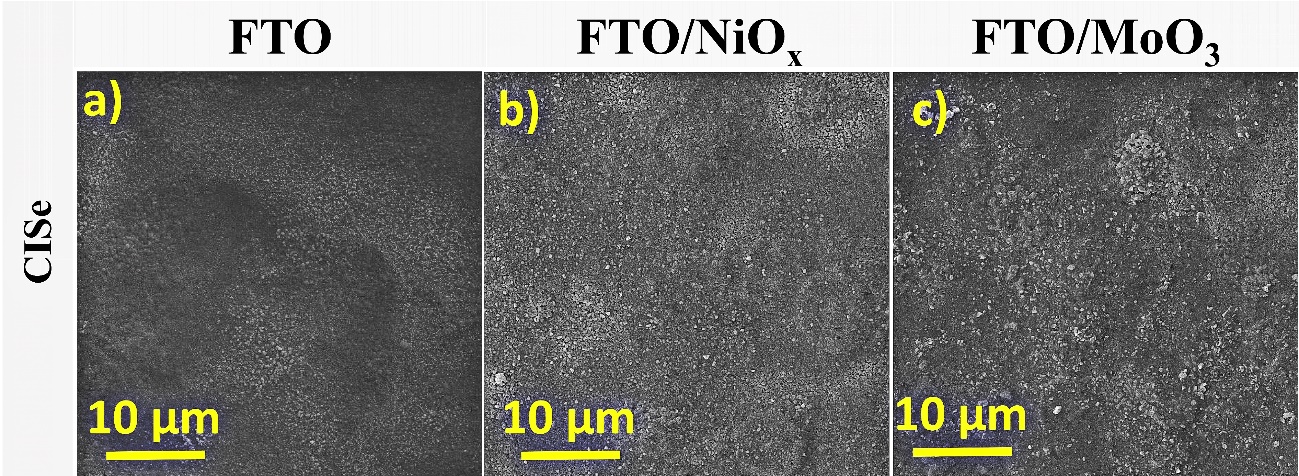


Figure S2: FESEM Surface micrograph of CISe films with various substrates; a) FTO,

b) FTO/NiO_x_, c) FTO/MoO_3_

Figure S3: EDS spectrum of bulk CISe films with the various substrate; a) FTO, b) FTO/NiO_x_, c) FTO/MoO_3_.

Table S1: Chemical composition of CISe films with the various substrate; FTO, FTO/NiO_x_ , and FTO/MoO_3_

determined by EDS.

| **Cu+In/S+Se** | **S+Se/In** | **Se/In** | **S/In** | **Cu/In** | **Sample** |
| --- | --- | --- | --- | --- | --- |
| 0.99 | 2.09 | 1.67 | 0.37 | 1.04 | ***FTO*** |
| 0.99 | 1.79 | 1.26 | 0.53 | 0.80 | ***FTO/NiO_x_*** |
| 0.90 | 1.96 | 1.52 | 0.44 | 0.77 | ***FTO/MoO_3_*** |


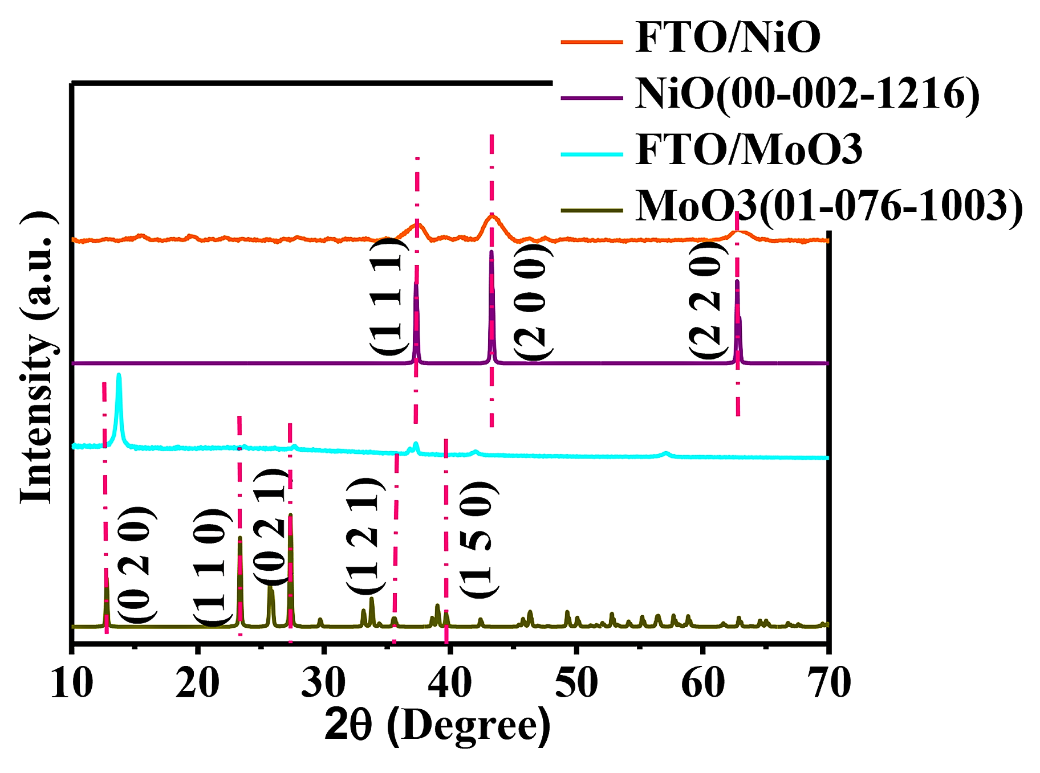


Figure S4: XRD pattern of NiO_x_ and MoO_3_ /glass substrates.
